# DNA Polymerase ζ without the C-Terminus of Catalytic Subunit Rev3 Retains Characteristic Activity, but Alters Mutation Specificity of Ultraviolet Radiation in Yeast

**DOI:** 10.3390/genes13091576

**Published:** 2022-09-02

**Authors:** Hollie M. Siebler, Jian Cui, Sarah E. Hill, Youri I. Pavlov

**Affiliations:** 1 Fred & Pamela Buffett Cancer Center, Eppley Institute for Research in Cancer, University of Nebraska Medical Center, Omaha, NE 68198, USA; 2 Department of Biology, Creighton University, Omaha, NE 68178, USA; 3 Department of Genetics, Cell Biology and Anatomy, University of Nebraska Medical Center, Omaha, NE 68198, USA; 4 Departments of Pathology and Microbiology, Biochemistry and Molecular Biology, University of Nebraska Medical Center, Omaha, NE 68198, USA

**Keywords:** UV mutagenesis, mutation spectra, pol ζ, TLS

## Abstract

DNA polymerase ζ (pol ζ) plays a central role in replicating damaged genomic DNA. When DNA synthesis stalls at a lesion, it participates in translesion DNA synthesis (TLS), which helps replication proceed. TLS prevents cell death at the expense of new mutations. The current model indicates that pol ζ-dependent TLS events are mediated by Pol31/Pol32 pol ζ subunits, which are shared with replicative polymerase pol δ. Surprisingly, we found that the mutant *rev3-*Δ*C* in yeast, which lacks the C-terminal domain (CTD) of the catalytic subunit of pol ζ and, thus, the platform for interaction with Pol31/Pol32, retains most pol ζ functions. To understand the underlying mechanisms, we studied TLS in normal templates or templates with abasic sites in vitro in primer extension reactions with purified four-subunit pol ζ versus pol ζ with Rev3-ΔC. We also examined the specificity of ultraviolet radiation (UVR)-induced mutagenesis in the *rev3-*Δ*C* strains. We found that the absence of Rev3 CTD reduces activity levels, but does not alter the basic biochemical properties of pol ζ, and alters the mutation spectrum only at high doses of UVR, alluding to the existence of mechanisms of recruitment of pol ζ to UVR-damaged sites independent of the interaction of Pol31/Pol32 with the CTD of Rev3.

## 1. Introduction

UV radiation (UVR) is a ubiquitous DNA-damaging mutagenic agent, and living cells possess multiple mechanisms of protection from its consequences [1,2,3]. UVR in the solar spectrum is a major cause of skin cancer [4,5,6]. UVR is divided into three wavelength groups, with varying degrees of effects on DNA [7,8]. This study utilized UVC (<280 nm) radiation, which is typically blocked by ozone; however, the ozone layer is being depleted in some areas and, thus, this deadly radiation can contribute to DNA damage [9]. UVC is often used to study UVR-induced mutagenesis because the major DNA lesions are the same as those resulting from UVA/UVB radiation, but are induced more efficiently [10]. The two most abundant DNA lesions induced by UVR are cyclobutane-pyrimidine dimers (CPDs) and 6,4 photoproducts (6-4PPs) [11]. Both lesions distort the DNA helix, impeding replication and transcription. CPDs comprise nearly 75% of UV lesions and are the most cytotoxic. 6-4PPs present a difficult template for DNA polymerases and have been shown to correlate with mutational hotspots better than CPDs [12].

When DNA repair does not occur before UVR-induced damage is encountered by the replication machinery, CPDs and 6-4PPs can cause replication fork arrest, double-stranded breaks, and, ultimately, cell death. Under these conditions, these lesions can be bypassed through recombination-like mechanisms involving the intact homologous strand [13] or by translesion DNA synthesis (TLS) utilizing specialized DNA polymerases (pols) [14,15,16]. The main TLS pols that act upon damage induced by UVR in yeast are pol η and pol ζ. Pol η is a member of the Y-family of DNA polymerases and is primarily involved in TLS events that lower the probability of mutations (frequently referred to as error-free events) [3,15,16]. It can facilitate efficient and relatively accurate bypass of CPDs, inserting two As across from abundant TT dimers in 99% of events [17,18]. The role of pol η in the bypass of 6-4PPs is controversial. Genetic studies have shown that pol η plays only a minor role in the bypass of these lesions [19,20], and it was shown that deletion of *RAD30* (encoding for yeast pol η) only decreased bypass of a single 6-4PP in a plasmid by 7.5% [21]. The absence of any of the three subunits of pol ζ Rev3, Rev7, or Pol32 leads to complete UVR immutability in yeast [22,23]; therefore, the most crucial role in converting UVR lesions to altered DNA sequences is attributed to pol ζ.

Pol ζ specializes in extension of distorted primer termini left by another polymerase [24]. It can also act as both the inserter and extender polymerase across some types of lesions. Pol ζ synthesizes DNA in vitro with low fidelity, and produces a characteristic mutational signature [25,26] that can be found in mutation spectra in vivo [27,28,29,30,31,32]. Part of this signature comprises multiple mutations in a row (called complex mutations), and these are generally attributed to template switches [33].

Pol ζ plays a key role in the error-prone bypass of UVR-induced lesions. It has been suggested that, in vivo, pol ζ plays a major role in bypassing 6-4PPs but a minimal role in bypassing CPDs, even though it can inaccurately bypass a CPD lesion in vitro [21,34]. This trend is opposite that of pol η. Pol ζ is most efficient in extending from distorted termini that result from nucleotides inserted by other polymerases [19]. Pol η and pol ζ may act together on these lesions, where pol η acts as the inserter and pol ζ acts as the extender [35,36]. When pol η is absent in yeast cells, mutagenesis induced by UVR is elevated, suggesting that pol ζ can take over bypass of these lesions [37]. More than 90% of UV-induced mutations, and nearly half of spontaneous mutations, in yeast are dependent on pol ζ [22].

**Figure 1:**
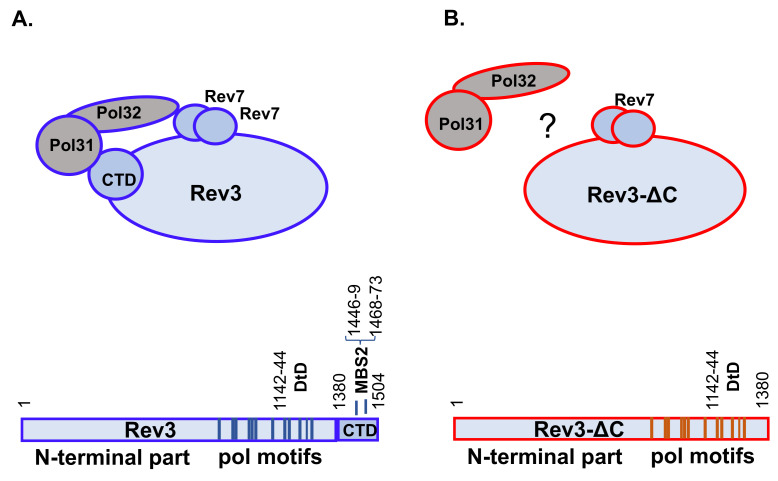
**Schematic structures of DNA pol ζ variants under study.** (**A**) Classic pol ζ_4_ (left image, blue contour) is a hetero-pentamer comprised of Rev3 (catalytic subunit, schematically shown under the structure), a dimer of two small accessory subunits of Rev7, and two subunits shared with DNA pol Δ, which are bound to the CTD of Rev3, Pol31, and Pol32. Below are the features marked on the amino acid sequence of Rev3: the N-terminal portion of unknown function, the polymerase motifs with the positions of the catalytic residues (DtD), and the C-terminal domain (CTD), along with the metal-binding motif (MBS2) that coordinates the Fe-S cluster. (**B**) Pol ζ_Rev3ΔC_ (red contour and outline of the catalytic subunit below) lacking the CTD, the hub thought necessary for interaction with Pol31/Pol32.

Pol ζ, previously thought to be a two-subunit enzyme [34], is actually a four-subunit enzyme containing the catalytic subunit Rev3 (for protein nomenclature, see [38]), consisting of the N-terminal region, catalytic core, and C-terminus domain (CTD) containing a 4Fe-4S cluster, and the accessory subunits Rev7, Pol31, and Pol32 [39,40,41,42] (Figure 1A). The latter two subunits are shared with the main replicative DNA pol δ. Pol ζ also binds another TLS pol, Rev1, forming a five-subunit complex [26]. In the four-subunit complex, Pol31 binds to the CTD of the catalytic subunit Rev3 and Pol32 binds to Pol31, while Rev7 binds directly to the core of Rev3 and Pol32 interacts with Rev7 [23,43], which has been confirmed by structural studies [44] (Figure 1A). The assembly of the whole complex is necessary for the proper function of pol ζ in mutagenesis, and yeast mutants with deletion of the *REV1*, *REV3*, *REV7*, or *POL32* genes are UV-immutable [22,43,45]. As deletion of *POL31* is lethal, it is only possible to assess its role in pol ζ-dependent mutagenesis by preventing its interaction with either Pol32 or with the CTD of Rev3 through amino acid changes. Mutations weakening the Pol31–Pol32 interaction severely reduce UV mutagenesis [46]. The main role in this interaction of the Rev3-CTD and Pol31 has been attributed to a 4Fe-4S cluster in Rev3. Strains with mutations leading to amino acid changes in this iron cluster-binding motif (MBS2 in Figure 1A, also called cysB) abolish the interaction and become UV-immutable [39,40,41,42]. Thus, the CTD of Rev3 plays an essential role in regulating pol ζ’s participation in TLS.

Given this documented role of the Rev3-CTD, it was surprising to discover that yeast strains with the *rev3-*Δ*C* deletion mutation encoding for a variant of Rev3 without the CTD—thus lacking the whole platform for the assembly of four-subunit pol ζ (Figure 1B), in contrast to point mutations affecting Fe-S binding, exhibit normal UVR mutagenesis at low doses and moderate defects in mutagenesis at higher doses [47]. Extending these studies, here we asked two additional questions, expanding our genetic work and introducing supporting biochemical analysis. First, we explored whether pol ζ with the Rev3-∆C catalytic subunit (named pol ζ_Rev3ΔC_) is catalytically active and retains the capability to bypass damaged DNA in vitro. Second, we investigated whether the specificity of UVR mutagenesis is changed in the *rev3-*∆*C* mutant to help further define the mechanism of mutagenesis by crippled pol ζ. We found that pol ζ_Rev3ΔC_ retains most properties of pol ζ, suggesting that it is recruited to TLS via a pathway independent of the Rev3-CTD. UVR mutation spectra in *rev3-*∆*C* strains differed at different doses, and generally showed severe defects in mutagenesis pathways leading to transversions, while being quite proficient in mutagenesis leading to transitions. The majority of mutations occurred in dipyrimidine sites, consistent with the dependence of mutagenesis on the two major types of UV-induced damage. Complex mutations, a hallmark result of the activity of the complete pol ζ complex, were not abrogated. Taken together, biochemical data and in vivo mutation-specificity analysis imply that the main properties of pol ζ are not be altered by the absence of the CTD (and, as a consequence, the absence of the 4Fe-4S cluster) of Rev3 and suggest that there are yet-to-be determined mechanisms compensating for the loss.

## 2. Materials and Methods

### 2.1. Nomenclature and Abbreviations

Ultraviolet radiation is abbreviated as UVR. Abasic sites are represented by the symbol “**θ**”. DNA polymerases are abbreviated as “pols”. Forms of pol ζ: 1) the four-subunit complex Rev3-Rev7-Pol31-Pol32 is denoted pol ζ_4_, 2) complexes with Rev3-ΔCTD are denoted pol ζ_Rev3ΔC_. The three-subunit complex Pol3exo-Pol31-Pol32 of the proofreading-deficient pol δ is denoted pol δ_exo-_. Mutasome = a complex of all proteins required for error-prone TLS.

### 2.2. Yeast Strains, Media, and Plasmids

DNA pol δ was overproduced as described previously [48]. For pol ζ purification, we used a similar system to that designed by P. Burgers: protease-deficient *Saccharomyces cerevisiae* yeast strain BJ2168 was transformed by plasmids pBL813 (*URA3 GAL1-10 REV3opt REV7opt*) and pBL347 (*LEU2 GAL1-10 POL31 7xHIS_POL32*) [41]. This strain overproduces all four subunits of pol ζ after induction by galactose. A variant of pBL813 encoding for CTD-less Rev3 (Rev3ΔC) was created in our laboratory by site-directed mutagenesis using the GenEdit kit (First Biotech, Kyoto, Japan). First, a fragment of codon-optimized *REV3*opt was amplified by PCR with primers cdel-SP1 5′TGTAAAACGACGGCCAGTGA and cdel-SP2 5′GACAAGATCCCAGACCCAGC, and the 3.1 kb HindIII-XhoI part of the PCR was cloned into a helper plasmid included in the kit. Site-directed mutagenesis was done using the primers cdel-OP1 5′TAAGCGAATTTCTTATGATTTATGA and cdel-OP2 5′AGAAGCTCTCTTAGACTTAACGA, which amplify the whole plasmid without the portion of *REV3opt* coding for the Rev3 CTD. After verification that the deletion was generated, the fragment was inserted back into pBL813. The plasmid encodes Rev7 and GST-Rev3-ΔCTD, lacking amino acids 1381–1504 of Rev3 (Figure 1).

UVR mutagenesis studies were performed in yeast strain 8C-YUNI101 (*MAT***a** *his7-2 leu2-3,112 ura3-Δ bik1*::*ura3-29RL trp1-1_UAG_ ade2-1_UAA_*) [49] and its derivatives, as described previously [47]. This strain is referred to as wild-type throughout the manuscript. The same strain with the deletion of the CTD of Rev3 is referred to simply as *rev3-*Δ*C*.

We used standard yeast media with modifications and appropriate carbon sources, as described previously [50]. Agar, yeast nitrogen base, yeast extract, peptone, glucose are from Formedium, Norfolk, UK. Other medium supplements are from Thermo Fisher, Waltham, MA, USA.

### 2.3. Polymerase Purification

Pol δ-5DV was purified as described previously [48]. For four-subunit pol ζ (pol ζ_4_), we followed published protocols with minor modifications [26,41]. Yeast cultures were grown in a selective medium (complete synthetic glucose lactic acid (SCGLA) medium lacking leucine and uracil (-leu-ura)) at 30 °C overnight with shaking (225 rpm), and yeast extract peptone glycerol lactic acid adenine (YPGLA) medium was added when the OD_660_ was between 1 and 2. At OD_660_ ≥ 3, galactose was added to induce protein expression, and cultures were incubated for another 12 h. A total of 200 g of yeast was collected by centrifugation (Avanti J-20 XP model from Beckman-Coulter, Pasadena, CA, USA), and resuspended in 3x lysis buffer (150 mM Hepes-KOH (all common reagents unless stated otherwise are from Millipore-Sigma, St. Louis, MO, USA, or Fisher Scientific, Waltham, MA, USA), pH 7.8, 90 mM K_2_HPO_4_/KH_2_PO_4_, pH 7.8, 8% glycerol, 7.5 mM sucrose, 0.03% Nonidet P-40, 0.15% Tween 20, 6 mM DTT, 900 mM KCl, 30 μM pepstatin A, 30 μM leupeptin, 7.5 mM benzamidine). Then, 200 g of yeast suspension was frozen by slowly pouring the suspension dropwise, with stirring, into liquid nitrogen and then stored at −80 °C. During the first step of purification, frozen cells were disrupted in liquid nitrogen in the Freezer Mill (Spex SamplePrep, Metuchen, NJ, USA) using the following parameters: precool—5 min; cycle—10 min; grinding—2 min; cool down—2 min. All further steps were carried out at 4 °C. Reagents were added to the lysate to the following final concentrations: phenylmethylsulfonyl fluoride (PMSF)—1 mM, glycerol—8%, then ammonium sulfate—0.15 M and Polymin P—0.45%. The mixture was stirred for 20 min to precipitate nucleic acids. The supernatant was collected by centrifugation at 18,000 rpm for 25 min. Then, 0.31 g/mL ammonium sulfate was added, and the solution was stirred for 15 min. A pellet was collected through centrifugation at 18,000 rpm for 25 min and resuspended in buffer A1 (50 mM Hepes-KOH, pH 7.4, 30 mM K_2_HPO_4_/KH_2_PO_4,_ pH 7.4, 8% glycerol, 2.5 mM sucrose, 0.01% Nonidet P-40, 0.05% Tween 20, 2 mM DTT, 300 mM KCl, 10 μM pepstatin A, 10 μM leupeptin, 2.5 mM benzamidine, 1 mM PMSF).

Next, 1 mL of glutathione Sepharose 4B resin (Cytiva, Marlborough, MA, USA) was added, and the mixture was gently agitated for 2 h. The resin was packed into a disposable column (BioRad, Hercules, CA, USA) and washed three times: once with 200 mL of buffer A1, once with 200 mL of buffer A2 (30 mM Hepes-KOH, pH 7.8, 30 mM K_2_HPO_4_/KH_2_PO_4,_ pH 7.8, 8% glycerol, 2.5 mM sucrose, 0.01% Nonidet P−40, 0.05% Tween 20, 1 mM DTT, 200 mM KCl, 10 μM pepstatin A, 0.5 mM PMSF, 5 mM MgCl_2_, 1 mM ATP), and once with 200 mL of buffer A3 (30 mM Hepes-KOH, pH 8.0, 30 mM K_2_HPO_4_/KH_2_PO_4,_ pH 8.0, 8% glycerol, 2.5 mM sucrose, 0.01% Nonidet P-40, 0.05% Tween 20, 1 mM DTT, 100 mM KCl, 10 μM pepstatin A, 0.5 mM PMSF). The protein was eluted with 4–5 stepwise washes with 2 mL buffer A3 containing 30 mM reduced L-glutathione. The GST tag in combined fractions was cleaved with PreScission protease (Cytiva) overnight. Then, the solution was diluted twofold with buffer E (30 mM Hepes-KOH, pH 7.4, 20 mM K_2_HPO_4_/KH_2_PO_4_, pH 7.4, 5% glycerol, 2.5 mM sucrose, 1 mM DTT, 200 mM KCL, 0.01% E10-C12, 0.5 mM PMSF) containing 10 mM imidazole and incubated with 2 mL Profinity™ IMAC Ni-charged resin (BioRad) for 1 h. The resin was washed with 200 mL buffer E with 20 mM imidazole. Protein was eluted with 500 μL of buffer E with 400 mM imidazole and dialyzed against buffer D (30 mM Hepes-KOH, pH 7.4, 200 mM NaCl, 8% glycerol, 1 mM DTT). The fraction was aliquoted in 30 µL and stored at −80 °C.

Purification of pol ζ_Rev3ΔC_ differed in the usage of the Mono S column using AKTA chromatography system (Amersham Biosciences, Amersham, UK) instead of metal-chelate affinity chromatography. Fractions after PreScission protease digest were loaded onto a 1 mL Mono S column (Cytiva) equilibrated and washed with buffer B1 (30 mM Hepes-KOH, pH 7.4, 10 mM K_2_HPO_4_/KH_2_PO_4_, pH 7.4, 5% glycerol, 2.5 mM sucrose, 1 mM DTT, 70 mM KCL, 0.01% E10-C12), eluted by step-gradient with buffer B2 (30 mM Hepes-KOH, pH 7.4, 10 mM K_2_HPO_4_/KH_2_PO_4_, pH 7.4, 5% glycerol, 2.5 mM sucrose, 1 mM DTT, 700 mM KCL, 0.01% E10-C12), and stored as described above.

### 2.4. DNA Substrates

For DNA polymerase activity assays, a synthetic 50-mer oligodeoxynucleotide with the sequence 5′-TTACTTTTCTCATCTTTTAATGGTATTGAC**C**CACGTCTGTGGTGTGTTTG-3′, complementary to nucleotides 91–140 of the *CAN1* gene, was used as the template. The same 49-mer oligodeoxynucleotide containing an abasic site (bold and underlined) was used as a damaged template. A 16-mer labeled with fluorescent dye Cy5 (5′-Cy5-CAAACACACCACAGAC-3′) served as a primer. Oligos were synthesized by IDT (Coralville, IA, USA)

Cy5-labeled primer and template strands (500 nM each) were annealed in 25 mM Tris-HCl, pH 7.5, and 150 mM NaAc by heating to 95 °C for 5 min and then slowly cooled to room temperature. Annealed DNA substrates were stored at −20 °C for up to 2 weeks.

**Figure 2:**
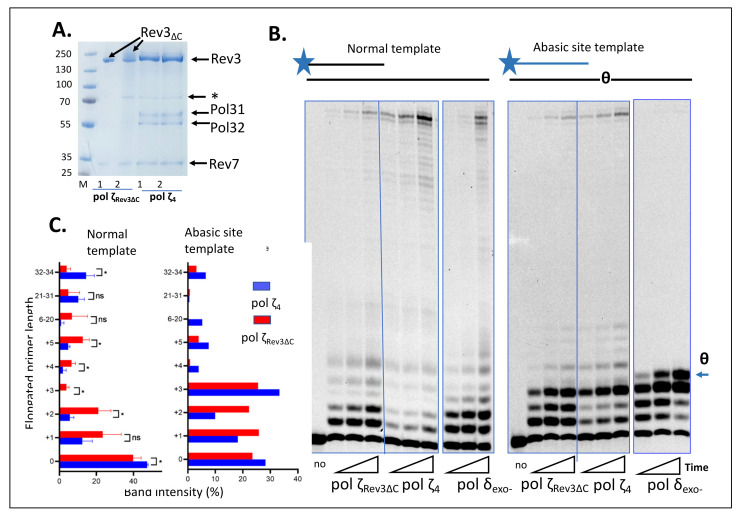
Polymerase ζ lacking C-terminal domain of Rev3 (pol ζ_Rev3ΔC_) was active and retained the ability to synthesize DNA on damaged templates similar to four-subunit pol ζ. (**A**) The appearance of pol ζ and pol ζ_Rev3ΔC_ (two independent preparations on nearby lanes) on Coomassie gel. M-Ruler Plus protein markers, apparent Mr on 4–15% PAAG, are in the left lane. * = chaperone band; “no” = no polymerase added. (**B**) Pol ζ_Rev3ΔC,_ like pol ζ_4_, could bypass abasic sites, albeit less efficiently. This activity was distinct from that of pol Δ, which could not elongate primer termini after the abasic site in the template (marked “θ” to the right of the gel). The pol ζ ratio to the substrate was 0.5, that of pol ζ_Rev3ΔC_ was 2, and that of pol Δ was 0.1. Reaction times were 5, 10, 25 min. “no”, no polymerase added to reaction. (**C**) Quantitative comparison of products of synthesis by pol ζ_Rev3ΔC_ (red) with pol ζ_4_ (blue) on normal template. *, *p* < 0.05, one-way ANOVA.

DNA polymerase activity assays with fluorescent primers were performed as described in [51,52]. In brief, assays were performed at 30 °C in 10 µL reaction mixtures containing 50 mM Tris-HCl, pH 7.5; 8 mM MgCl_2_; 100 µg/mL BSA; 1 mM DTT; 100 μM each of dATP, dCTP, dGTP, and dTTP; and 50 nM annealed DNA substrate. Polymerase concentration and reaction time are indicated in the legend off Figure 2. After incubation, reactions were terminated with 10 μL denaturing gel loading buffer containing 95% formamide, 10 mM ethylenediaminetetraacetic acid (EDTA), and 0.125% Orange G. After incubation at 95 °C for 5 min, the reaction mixtures were loaded onto a 12.5% denaturing polyacrylamide gel (7 M urea). Gel electrophoresis was performed in ThermoFisher Owl S3S box in buffer with 85 mM Tris, 85 mM boric acid, and 2 mM EDTA at 50 W for 2 h and scanned with a Typhoon™ FLA 9500 biomolecular imager (Cytiva). The pattern of DNA synthesis was determined by analyzing each extended band intensity in comparison to the intensity of the unextended primer in ImageQuant TL 8.2 analysis software (Cytiva).

### 2.5. Determination of Survival and Induced Mutation Frequencies Using Can^r^ Assay

Experiments were performed essentially as in [47]. Yeast strains were grown for two days at 30 °C in 5 mL of yeast extract peptone dextrose medium with 60 mg/L adenine and uracil (YPDAU) with shaking. Cells were pelleted at 1000 g in a Beckman Model TJ-6 centrifuge for 2 min and re-suspended in 1 mL of sterile water. Cells were appropriately diluted, and 100 µL aliquots were plated on synthetic complete (SC) medium for estimation of viability; 50–100 µL of undiluted cells were plated on SC-arg medium containing 60 mg/L of L-canavanine (Can). Plates were irradiated with UVC within 10 min of plating. After three days of growth at 30 °C, colonies on SC plates were counted, and survival was calculated by dividing the number of colonies at each UV treatment by the number of colonies without exposure. After five days of growth, colonies on SC + Can plates were counted, and the mutation frequency was calculated by dividing the number of colonies on SC + Can plates at each UV dose by the number of colonies on the SC plate at the same dose (the SC colony count was first multiplied by the dilution factor). The induced Can^r^ mutant frequency was calculated by subtracting the spontaneous frequency (without treatment) from the mutant frequency for each UV dose [20]. All data points are averages of at least four independent trials, with duplicates of each sample in each trial.

### 2.6. Analysis of Mutational Spectra

Cells were plated and irradiated as described above for the Can^r^ assay. Then, independent colonies were collected from each strain (wild-type and *rev3-*∆*C*) at each dose and were colony-purified by streaking them out on SC + Can plates (one colony per quarter-plate). One colony was picked from each quarter, genomic DNA was isolated, and the *CAN1* gene was amplified with PCR using DreamTaq Green DNA Polymerase (Thermo Scientific, Waltham, MA, USA) and the primers CAN1ext-F2 and CAN1ext-R2 (F2—TCTTCAGACTTCTTAACTCC, R2—ATAGTAAGCTCATTGATCCC) or CAN1ampF 5′TTCTGTGTGGTTTCCGGGTGAG and CAN1ampR 5′ TGCTTCTACTCCGTCTGCTTTCTTTTC using SimpliAmp Thermal Cycler (Applied Biosystems/ThermoFisher, Waltham, MA, USA). The PCR products were purified using PEG 8000 precipitation [53] and sent to Genescript (Piscataway, NJ, USA) or McLab (San Francisco, CA, USA) for sequencing using the primers CAN1seq520F: TGGTTTTCTTGGGCAATCAC, CAN1seq699R: GATGGAAGCGACCCAGAAC, and CAN1seq1026F: AGTTCCATACAATGACCCTA. DNA sequences were then analyzed using Geneious Pro software v.5-v.R10 (Biomatters, Aukland, New Zealand).

### 2.7. Statistical Analyses

Survival and mutation frequencies are represented by means ± standard deviations. Unpaired two-tailed Student’s *t*-tests were used to test the difference in survival and induced mutant frequency between wild-type and *rev3-*Δ*C*. The χ^2^ test of independence was applied to determine the differences in the mutation spectra between wild-type and *rev3-*Δ*C,* as well as the mutation spectra in dipyrimidine (DP) sites. Fisher’s exact test was used to compare the mutation spectra when the sample sizes were small. All analyses were performed using the open source software R. *P* < 0.05 was considered statistically significant.

## 3. Results

### 3.1. Pol ζ_Rev3ΔC_ Shows Hallmark Characteristics of the TLS Polymerase pol ζ_4_, but Has Lower Activity In Vitro

The results of the purification of four-subunit pol ζ and a variant that lacks the C-terminal domain of the catalytic subunit, Rev3-ΔC, are shown in Figure 2A. The catalytic subunit, Rev3, was N-terminally tagged by GST in the overexpression constructs, which allowed affinity purification of protein complexes tightly bound to Rev3. GST was subsequently cleaved from Rev3 (see the Section 2). Preparations of pol ζ_4_ showed all four expected subunits (Figure 1A and Figure 2A), while preparations of pol ζ_Rev3ΔC_ did not show Pol31/Pol32 subunits, confirming that their interaction was severely impaired by the CTD deletion preventing co-purification (Figure 1B and Figure 2B). Despite the lack of the subunits thought essential for pol ζ activity [39,40,41], preparations of pol ζ_Rev3ΔC_ were catalytically active in vitro, though higher concentrations were required to get synthesis to the end of the template (Figure 2B). This was consistent with our previous in vivo data [47]. Importantly, pol ζ_Rev3ΔC_ was able to bypass and extend past an abasic site similar to pol ζ_4,_ in stark contrast to replicative pol δ. The distribution of extended products showed an elevated tendency of pol ζ_Rev3ΔC_ to terminate synthesis prematurely, especially before (position +3) the abasic site (Figure 2C).

### 3.2. Rev3-∆C ιs Most Proficient at Participating in the Formation of Transition Mutations In Vivo

**Figure 3:**
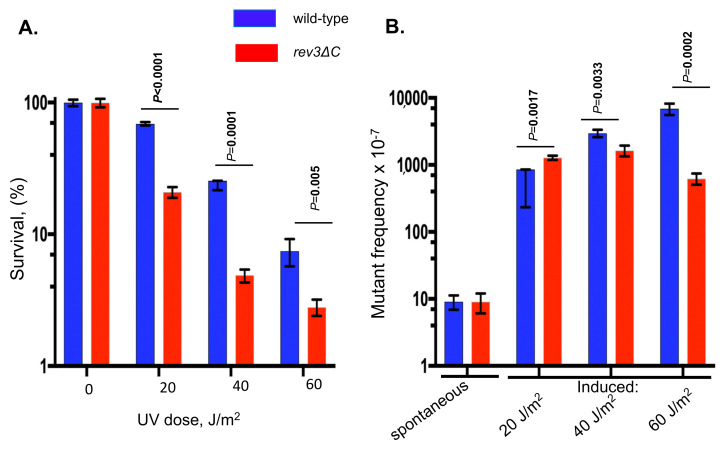
Survival (**A**) and mutagenesis levels (**B**) in UV-irradiated wild-type and *rev3-*∆*C* strains in comparison to untreated cells. Values are means from four independent experiments. Wild-type, blue ●; *rev3*Δ*C*, red ■. Data were collected and analyzed as described in the Section 2. The significance of the difference between wild-type and *rev3-*Δ*C* strains was evaluated by a two-tailed unpaired Student’s *t*-test.

Additional tests confirmed our previous observations [47] on the increased sensitivity of *rev3-*∆*C* strains to UVR-induced killing and proficiency in UVR-induced mutagenesis. The *rev3-*∆*C* strain was up to 50% more sensitive to UV than the wild-type strain (Figure 3A). UVR dramatically increased (two orders of magnitude) *can1* mutant frequencies in both strains, but, at higher doses, the frequency of induced mutants in *rev3-*∆*C* was profoundly lower compared to the wild-type strain, though still far above the level of spontaneous mutations (Figure 3B). The magnitudes of the effects were consistent with a previous study [47].

To explore further than previously, we determined *can1* mutation spectra at three different doses to accurately reflect the dose-dependent changes in the *rev3-*∆*C* strain (Table 1 and Tables S1 and S2 with more details on the statistical analysis). Mutations are shown mapped to the *CAN1* gene in Figure S1A–C. For the analysis, mutations were divided into the major classes of base substitutions plus insertions/deletions (indels) and complex mutations (Table 1 and Table S1).

#### 3.2.1. Dose of 20 J/m^2^

**Figure 4:**
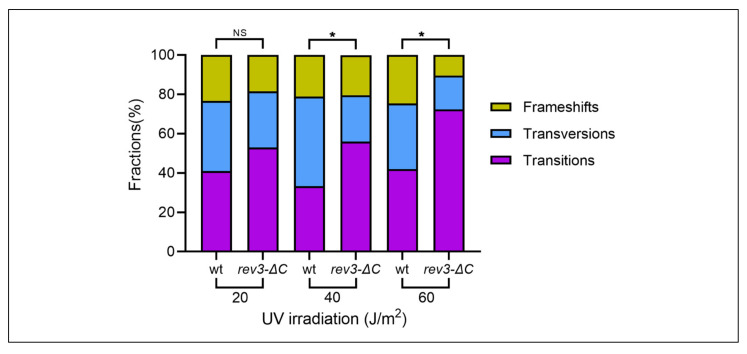
The proportion of transversion mutations progressively decreased with increasing UV- dose in the mutation spectra of *rev3-*∆*C*. Breakdown of proportions of different classes of mutations detected at 20 J/m^2^, 40 J/m^2^, and 60 J/m^2^. * *p* < 0.05.

At the lowest dose used, *rev3-*∆*C* showed small increases over the wild-type in the frequency of all types of mutations, except for complex mutations; however, differences in the overall proportions of mutations of all types were not statistically significantly different from the wild-type (Table 1, Figure S2A). The proportions of transition and transversion mutations were the same (Figure 4, two left bars). Approximately one-fifth of all mutations were indels. The spectra in the wild-type and *rev3-*∆*C* strains were similar, with congruency between several hot spots: single base substitutions at positions 452, 527, 938, 1471, and 1477 and coincidence of complex mutations at position 723 (Figure S1A). The results suggest that, at this dose, pol ζ_Rev3ΔC_ was fully functional in its transactions in UVR-induced mutagenesis.

#### 3.2.2. Dose of 40 J/m^2^

The intermediate dose caused a 3.5-fold further increase in mutation frequency in the wild-type strain compared to the previous dose, but *rev3-*∆*C* strains showed only a subtle increase, which led to a twofold difference in total induced mutant frequency compared to the wild-type (Figure 3, Table 1, last row). At this dose, the difference between the two strains’ mutational spectra started to appear (Figure 4**,** two middle bars). The *rev3-*∆*C* spectrum displayed 3.5-fold lower frequencies of transversions and a 1.9-fold decrease in frameshift mutations (Table 1, Figure S2B). Interestingly, the frequency of transitions was unaffected compared to the wild-type strain. As a result, the proportions of different classes of mutations were different between the strains (Figure 4). Mutations are shown mapped to the *CAN1* gene in Figure S1B. The overlap in the distributions of mutations between wild-type and *rev3-*∆*C* was less obvious than at 20 J/m^2^. We could see cases of coincidences in mutations at positions 527, 938, and 1234. Positions 930–931 showed similar complex mutations in both strains. Another interesting similarity was the occurrence of similar mutations at position 1477 in both spectra and doses so far discussed. We inferred from the results at this dose that pol ζ_Rev3ΔC_ has limitations in the processes, leading to all types of mutations except transitions.

#### 3.2.3. Dose of 60 J/m^2^

At the highest dose, *rev3-*∆*C* exhibited an 11-fold reduction in overall induced mutant frequency (the frequency of UVR-induced mutations still exceeded spontaneous mutation frequency by 50-fold (Figure 3); the strain was UVR-mutable) and exhibited much lower frequencies for all types of mutations compared to the wild-type (Table 1, Figure S2). The defect in transitions was less severe at 6.4-fold. Transversions decreased by 22-fold, frameshifts decreased by 25-fold, and complex mutations decreased by 89-fold (Table 1 and Table S1). At this dose, CG-to-TA transition mutations dominated the spectrum in *rev3-*∆*C* (71% of all mutations, Figure 4). Mutations are shown mapped to the *CAN1* gene in Figure S1C. We observed a C-to-T transition mutation hotspot at position 527 in both strains, which was the same as in the 20 J/m^2^ spectra. Furthermore, positions in the vicinity of 1478 also had several mutations, even complex mutations, in the *rev3-*∆*C* strain. Overall, the *rev3-*∆*C* strain was most proficient in UVR-induced transitions. We inferred that, at this dose, pol ζ_Rev3ΔC_ was partially impaired in transactions leading to UVR-induced mutations, but the ability to extend mismatches leading to transitions was less affected.

### 3.3. Pol ζ_Rev3ΔC_ Participates in Creating pol ζ-specific Complex Mutations with Reduced Efficiency

Nearly all UVR-induced mutations are dependent on pol ζ, and its mutational signature has been studied in vitro and in vivo (see the Introduction). One of the prominent mutagenesis features dependent on pol ζ is the formation of complex mutations, defined here as multiple mutations in the same clone within ten base pairs. Pol ζ_Rev3ΔC_ was capable of supporting pathways leading to complex mutations, but these mutations were found at lower frequencies than with the wild-type for all doses studied (see Table S1 and Figure S1).

### 3.4. The Majority of Mutations in Wild-type and rev3-∆C Strains Occur in Dipyrimidine (DP) Sites

**Figure 5:**
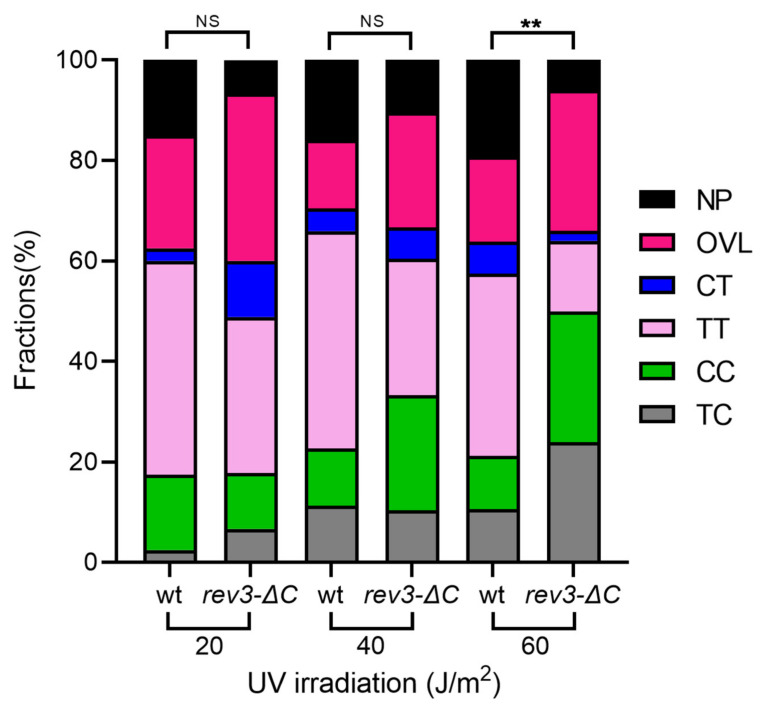
The proportions of mutations at di-pyrimidine sites in the *rev3-*∆*C* strain differed from the wild-type only at the highest dose. The *rev3-*∆*C* spectrum at 60 J/m^2^ showed a higher proportion of mutations in CC sites and a lower proportion in NP sites compared to the wild-type. NP, non-dipyrimidine sites; OVL, overlapping sites (e.g., TCT with mutated C); CT, 5′CT; TT, 5′TT; CC, 5′CC; TC, 5′ TC. ** = *p* < 0.01, NS = non-significant.

UVR primarily induces CPDs and 6-4PPs, both of which occur in DP sequences. Correspondingly, the majority (up to 90%) of mutations induced by UVR occur in these DP sites. The percentages of mutations found in DP sites versus non-DP sites were compared between wild-type and *rev3-*∆*C* strains, excluding complex mutations. In both strains, mutations predominantly occurred in DP sites, and there was no statistical difference between the two strains (Table 2). This result suggests that pol ζ_Rev3ΔC_ can participate in transactions leading to mutations during di-pyrimidine lesion bypass similarly to pol ζ4. The *rev3-*∆*C* strain showed a significant decrease in the proportion of TT sites mutated and an increase in the proportion of CC sites mutated at 60 J/m^2^ compared to the wild-type (Figure 5). It is possible that the activity of pol ζ_Rev3ΔC_, when lesions are abundant, is not equally sufficient for different types of UVR lesions.

## 4. Discussion

Given the multi-faceted role of pol ζ in mutagenesis and carcinogenesis, it is essential to understand the mechanism, and the precise roles and functions, of all subunits of pol ζ in transactions on damaged DNA. The protein complex performing TLS is called the mutasome. The recruitment of pol ζ may occur through several partially overlapping mechanisms dependent on protein–protein interactions and regulated by Fe-S clusters in pols. We centered our study on a yeast mutant that lacked the C-terminal domain (CTD) of Rev3 and, thus, the crucial interaction platform for Pol31/Pol32, which were thought to be required for pol ζ function (Figure 1). Breaking the current paradigm in TLS, the strain with crippled pol ζ had almost normal UV-induced mutagenesis [47] (Figure 3), pointing to the void in our understanding of pol ζ and the existence of a backup mechanism for its recruitment. Due to the similarities in the genetic processes and conservation of genes encoding for pols in yeast and human cells, it is likely that analysis of yeast mutants would provide new information on the mechanisms of pol ζ-assisted replication relevant to the onset and cure of human disease. This knowledge could help lead to the development of new dual-target approaches to the inhibition of TLS (in addition to the inhibition of REV3L/REV7/REV1 interactions), a promising anticancer target [54], where not one but two pathways of recruitment of pol ζ would be inhibited simultaneously.

In this study, we asked whether (a) Pol ζ_Rev3ΔC_ possesses the same basic biochemical properties as wild-type pol ζ, and (b) the Pol ζ variant present in the *rev3-*∆*C* mutant strain is deficient in participation in all types, or only certain types, of mutations in response to UVR. Comparing the biochemical properties of the two forms of pol ζ and the UV-induced mutational spectra between wild-type and *rev3-*∆*C* strains further defined the role of the CTD of Rev3 during TLS. We found that Pol ζ_Rev3ΔC_ behavior in vitro in normal and damaged templates was similar to that of pol ζ, but the overall activity was reduced. The spectra of UVR-induced mutations in the *rev3-*∆*C* strain showed a bias toward transition mutations. There was a milder defect with respect to transitions than transversions/frameshifts, and a higher UVR dose was needed to elicit this effect.

### 4.1. Biochemical Properties of pol ζ_Rev3ΔC._

We purified the complex from yeast strains overexpressing *GST-rev3-*∆*C, REV7, POL31*, and *POL32,* the system described for wild-type pol ζ_4_ [41] (Figure 2A). The main feature of the system is the use of the GST tag for affinity chromatography in one of the first steps of purification (note that the GST tag was cleaved after the initial pulldown of the GST-tagged catalytic subunit; see the Section 2). The protocol resulted in robust purification of the four-subunit pol ζ_4_ (Figure 2A). In the case of pol ζ_Rev3ΔC_, only two bands, corresponding to Rev3-ΔC and Rev7, were seen on the gel, confirming that the deletion of the CTD of Rev3 led to an inability to support strong interactions with Pol31/Pol32 subunits that could withstand conditions of purification. Despite the deficit of Pol31/32, Pol ζ_Rev3ΔC_ retained substantial DNA polymerase activity and had the ability to synthesize DNA on a template with an abasic site (Figure 2B,C), which was quite different from the inability to bypass abasic sites in the genuine two-subunit pol ζ_2_, purified from a strain with a deletion of the *POL32* gene [41].These results suggest that the characteristic conformation of the active site of Rev3 [55] was unaltered and functional. We hypothesize that the Rev3 CTD in pol ζ_2_ without Pol31/32 either adopted a conformation that was inhibitory for pol reaction or became purified as an inactive complex, whereas the Rev3 CTD without proper partners was misfolded; this constraint was bypassed in Pol ζ_Rev3ΔC_. Another possibility is that the preparation of pol ζ_Rev3ΔC_ contained various unknown minor accessory proteins (not visible in the Coomassie gel) that were sufficient to support the activity of the complex. The complex was purified from a strain that overexpressed components of pol ζ_4_ but had natural levels of all other replication proteins. A candidate protein was Rev1, which can interact with Rev7 and Pol32 [44,56,57,58] and, thus, could have brought the necessary components to the pol ζ_Rev3ΔC_ complex. In this scenario, we should assume different interactions of Rev1 within the pol ζ_Rev3ΔC_ and pol ζ_5_ complexes due to the presence of Rev1 in inhibiting the pol ζ_4_-Rev1 five-subunit complex [26]. The near-normal (at least at low or moderate levels of DNA damage) participation of Pol ζ_Rev3ΔC_ in UVR-induced mutagenesis indicated that the natural cell environment effectively compensated for the absence of the Rev3 CTD. It is possible that the high level of UVR mutagenesis in *rev3-*∆*C* was a result of a combination of the mechanisms discussed above.

### 4.2. UVR Mutation Spectra in rev3-∆C Strains

Studies of mutational spectra can provide a wealth of information that cannot be discerned by simply measuring rates of mutagenesis in cells [19,59,60]. Mutational spectra have been examined in the p53 gene in human skin cancers, which are primarily caused by UVR ([61]; reviewed in [60,62]). Most mutations in p53 in skin cancers are CG-to-TA transitions (mainly C-to-T), and 90–96% of all base substitutions occur in dipyrimidine sites. This well-known UV signature is consistent with the fact that UVR primarily induces CPDs and 6-4PPs, which occur at these sites.

It has been shown that mutational spectra from skin tumors are very similar to the spectra observed in UVR targets in model systems, such as yeast, but differ from spectra described in other cancers (discussed in [63]). The UVR spectrum in yeast transformed by an irradiated vector containing human p53 cDNA was not significantly different from that found in patient non-melanoma skin tumors [63], highlighting the fact that yeast is a suitable model to study cancer-relevant UVR mutagenesis.

In yeast, the *CAN1* gene is often used to study such mutational spectra (discussed in [19,20]). This system is able to detect all types of base-pair substitutions, deletions, insertions, and complex mutations. UVC-induced spectra in *CAN1* consist predominantly of base-pair substitutions and tandem and non-tandem complex mutations ([20]; discussed in [19]). These complex mutations are a signature of pol ζ [27,29,64,65]. Mutation spectra presented here for both strains share many features with the yeast *CAN1* spectra previously reported in the literature (discussed in [19]).

UV spectra were examined at three doses to accurately characterize the *rev3-*∆*C* strains (Table 1, Figure S1). Compared to the wild-type strain, this strain showed no statistically significant changes in the levels or distribution of mutations at the lowest UVR dose. At an intermediate dose, it showed a decreased ability to form transversion and indel mutations. At the highest dose, *rev3-*∆*C* exhibited a reduction in overall induced mutant frequency and lowered all types of mutations compared to the wild-type; however, the level of induced mutagenesis remained high, exceeding the level of spontaneous mutations by almost two orders of magnitude (Figure 3B). The decrease seen in the overall induced mutant frequencies with the increase in UVR doses in *rev3-*∆*C* strains could have been due to several reasons.

One reason could be the increased number of lesions, which a polymerase with lower activity would not be able to process. It is also plausible that lesions or sequence contexts where transversions usually form may not be efficiently bypassed by Pol ζ_Rev3ΔC_. Crippled pol ζ might have difficulties inserting bases resulting in transversions compared to the wild-type and/or it may be less efficient in extending transversions inserted by other polymerases, such as Pol η. Pol ζ_Rev3ΔC_ may be recruited less efficiently to lesions where transversions usually arise. It is conceivable that Pol ζ_Rev3ΔC_ may have less difficulty with transitions, as these involve the substitution of structurally similar bases (same numbers of rings) [55]. Transversions would distort the double helix of DNA, posing difficulties for both insertion and extension. It is formally possible that pol ζ_Rev3ΔC_ is an inferior enzyme, so only the mutations that are easy to produce/extend remain (transitions). Another possibility is that Pol ζ_Rev3ΔC_ is more proficient at bypassing one of the two major types of UV lesions. It will be essential in the future to determine whether the regulatory role of the CTD is in recruitment to DNA or whether it affects catalysis. Without a solved structure showing Rev3 with the CTD in the full Pol ζ_5_ complex, it is not clear where the domain lies relative to the catalytic residues or DNA binding site. Deamination of C residues in photoproducts [66] could also play a role in this process. If it takes more time for mutations to be addressed by pol ζ_Rev3ΔC_, this could allow time for the C residues involved to be deaminated to U, resulting in an A incorporation.

A prominent signature of pol ζ in UVR mutagenesis is the formation of complex mutations [20]. These can occur through a template-switching mechanism [33] or when pol ζ produces errors/mutations during the extension of sequences downstream of the lesion site. They are defined here as multiple mutations in the same clone within 10 base pairs, although it has been shown that stretches synthesized by pol ζ can be up to several hundred nucleotides in length [32]. In our strains, pol ζ_Rev3ΔC_ was able to participate in the formation of these complex mutations (Table S1, Figure S1), providing additional evidence for the functional proficiency of the enzyme in vivo.

Most UVR-induced mutations in wild-type and *rev3-*∆*C* strains were found in DP sites, consistent with the fact that most lesions caused by UVR are CPDs or 6-4PPs (Table 2). 6-4PPs are usually formed preferentially at CC and CT sites, whereas CPDs are formed at TT sites [61]. We observed that a higher proportion of total mutations were found in CC sites, and a higher proportion of total CC sites and a lower proportion of total TT sites were mutated in *rev3-*∆*C* at 60 J/m^2^ compared to the wild-type (Figure 5). Additionally, there was an increase in the proportion of mutations found at CC and CT sites and in the proportion of total CC/CT sites mutated at 60 J/m^2^ in *rev3-*∆*C* compared to lower doses. These data may reflect a stronger role for Pol ζ_Rev3ΔC_ in bypassing 6-4PPs (and deficiency in CPD bypass), especially at high doses of UVR.

Finally, we would like to emphasize that the complete absence of the domain of pol ζ necessary for binding the Fe-S cluster did not abolish the polymerase transactions necessary for TLS and induced mutagenesis, arguing against the involvement of electron transfer signaling in the regulation of pol ζ-assisted events hypothesized for the other members of B-family, primase-pol α, pol δ, and pol ε [67,68,69].

## Figures and Tables

**Table 1:** Differences in mutation specificity in wild-type and *rev3-*Δ*C* strains.

Mutation Type *	UVR dose (J/m^2^)								
	20			40			60		
	Wild-Type	*rev3-*Δ*C*	*p*-Value ^#^	Wild-Type	*rev3-*Δ*C*	*p*-Value ^#^	Wild-Type	*rev3-*Δ*C*	*p*-Value ^#^
**transitions**									
AT to GC	6	6		6	10		8	**1** **^#^**	0.0386
GC to AT	17	20		13	**23** **^#^**		21	**41** **^#^**	<0.0001
Total	23	26		19	**33** **^#^**		29	**42** **^#^**	0.0007
Rate **	3.5	6.8		9.9	9.1		29.0	4.5	
Fold decrease ^##^		0.5			1.1			6.4	
**Transversions**									
AT to TA	18	14		19	**8** **^#^**	0.0206	15	6	
Other	2	0		7	6		8	4	
Total	20	14		26	**14** **^#^**	0.0132	23	**10** **^#^**	0.0445
Rate **	3.0	3.7		13.6	3.9		24.0	1.1	
Fold decrease ^##^		0.8			3.5			22.3	
**Frameshifts**									
Deletions	10	8		10	9		16	**5** **^#^**	0.0322
Insertions	3	1		2	3		1	1	
Total	13	9		12	12		17	**6** **^#^**	0.0407
Rate **	2.0	2.4		6.2	3.3		16.0	0.6	
Fold decrease ^##^		0.8			1.9			24.8	
Total changes	56	49		57	59		69	58	
Total rate **	8.5	12.8		29.7	16.4		69.0	6.25	
Fold decrease ^##^		0.7			1.8			11.1	

*—Substitutions found in mutants double tandem mutants are counted as individual single base substitution. **—Induced mutant frequency × 10^−5^. ^#^—if no value in a cell = non significant. Exact values are given only for statistically significant difference in proportion of mutations (in **BOLD**), all values are in Supplementary Table S2. ^##^—Fold decrease of induced mutant frequency in *rev3-*Δ*C* comparing to wild-type.

**Table 2:** UVR- induced mutations occur predominantly in dipyrimidine (DP) sites in wild-type and *rev3-*∆*C* *.

DP Site	UVR Dose, J/m^2^					
	20		40		60	
	Wild-Type	*rev3-*Δ*C*	Wild-Type	*rev3-*Δ*C*	Wild-Type	*rev3-*Δ*C*
5′CC	6	5	5	11	5	13
5′CT	1	5	2	3	3	1
5′TC	1	3	5	5	5	12
5′TT	17	14	19	13	**17**	**7** ^#^
OVL	9	15	6	11	8	14
Total in DP sites (%)	34 (85%)	42 (93%)	37 (84%)	43 (86%)	38 (81%)	47 (94%)
Non-DP	6	3	7	5	9	3
Total changes	40	45	44	48	47	50

* Complex mutations excluded. ^#^—the only pair that was significantly different (*p* = 0.0177) between the wild-type and *rev3-*∆*C* strains is in bold, Fisher’s exact test. OVL—overlapping sites, e.g., TCT, where mutated base is “C”.

## Data Availability

Yeast strains and plasmids created in this work are available upon request.

## Supplementary Materials

The following supporting information can be downloaded at: https://www.mdpi.com/article/10.3390/genes13091576/s1, Figure S1: Mutational spectra of wild-type and *rev3-*∆*C* mapped to *CAN1* gene, Figure S2: UV dose-dependent changes in frequencies of transition, transversion, and indel mutations in the wild-type and *rev3-*∆*C* strains, Table S1: Differences in classes of mutations in wild-type and rev3-ΔC strains. Table S2: Differences in mutation spectra in wild-type and rev3-ΔC strains.
