# Supplementary material for: DNA Polymerase ζ without the C-Terminus of Catalytic Subunit Rev3 Retains Characteristic Activity, but Alters Mutation Specificity of Ultraviolet Radiation in Yeast
Source: Genes (Basel). 2022 Sep 2;13(9):1576. doi: 10.3390/genes13091576 (PMC9498848; doi:10.3390/genes13091576)
Supplement: Supplementary file 1 [file genes-13-01576-s001.zip › Suppl Figure S1.pdf]

A.

```
1     ATGACAAATT CAAAAGAAGA CGCCGACATA GAGGAGAAGC ATATGTACAA TGAGCCGGTC ACAACCCTCT TTCACGACGT TGAAGCTTCA
91    CAAACACACC ACAGACGTGG GTCAATACCA TTAAAAGATG AGAAAAGTAA AGAATTGTAT CCATTGCGCT CTTTCCCGAC GAGAGTAAAT
181   GGCGAGGATA CGTTCTCTAT GGAGGATGGC ATAGGTGATG AAGATGAAGG AGAAGTACAG AACGCTGAAG TGAAGAGAGA GCTTAAGCAA
271   AGACATATTG GTATGATTGC CCTTGGTGGT ACTATTGGTA CAGGTCTTTT CATTGGTTTA TCCACACCTC TGACCAACGC CGGCCCAGTG
361   GGCGCTCTTA TATCATATTT ATTTATGGGT TCTTTGGCAT ATTCTGTCAC GCAGTCCTTG GGTGAAATGG CTACATTCAT CCCTGTTACA
451   TCCTCTTTCA CAGTGTTCTC ACAAAGATTC CTTTCTCCAG CATTTGGTGC GGCCAATGGT TACATGTATT GGTTTTCTTG GGCAATCACT
541   TTTGCCCTGG AACTTAGTGT GTTGGCCAA GTCATTCAAT TTTGGACGTA CAAAGTTCCA CTGGCGGCAT GGATTAGTAT TTTTTGGGTA
631   ATTATCACAA TAATGAACTT GTTCCCTGTC AAATATTACG GTGAATTCGA GTTCTGGGTC GCTTCCATCA AAGTTTTAGC CATTATCGGG
721   TTTCTAATAT ACTGTTTTTG TATGGTTTGT GGTGCTGGGG TTACCGGCCC AGTTGGATTC CGTTATTGGA GAAACCCAGG TGCCTGGGGT
811   CCAGGTATAA TATCTAAGGA TAAAAACGAA GGGAGGTTCT TAGGTTGGGT TTCCTCTTTG ATTAACGCTG CCTTCACATT TCAAGGTACT
901   GAACTAGTTG GTATCACTGC TGGTGAAGCT GCAAACCCCA GAAAATCCGT TCCAAGAGCC ATCAAAAAAG TTGTTTTCCG TATCTTAACC
991   TTCTACATTG GCTCTCTATT ATTCATTGGA CTTTTAGTTC CATACAATGA CCCTAAACTA ACACAATCTA CTTCCTACGT TTCTACTTCT
1081  CCCTTTATTA TTGCTATTGA GAACTCTGGT ACAAAGGTTT TGCCACATAT CTTCAACGCT GTTATCTTAA CAACCATTAT TTCTGCCGCA
1171  AATTCAAATA TTTACGTTGG TTCCCGTATT TTATTTGGTC TATCAAAGAA CAAGTTGGCT CCTAAATTCC TGTCAAGGAC CACCAAAGGT
1261  GGTGTTCCAT ACATTGCAGT TTTCGTTACT GCTGCATTTG GCGCTTTGGC TTACATGGAG ACATCTACTG GTGGTGACAA AGTTTTCGAA
1351  TGGCTATTAA ATATCACTGG TGTTGCAGGC TTTTTTGCAT GGTTATTTAT CTCAATCTCG CACATCAGAT TTATGCAAGC TTTGAAATAC
1441  CGTGGCATCT CTCGTGACGA GTTACCATTT AAAGCTAAAT TAATGCCCGG CTTGGCTTAT TATGCGGCCA CATTTATGAC GATCATTATC
1531  ATTATTCAAG GTTTCACGGC TTTTGCACCA AAATTCAATG GTGTTAGCTT TGCTGCCGCC TATATCTCTG TTTTCCTGTT CTTAGCTGTT
1621  TGGATCTTAT TTCAATGCAT ATTCAGATGC AGATTTATTT GGAAGATTGG AGATGTCGAC ATCGATTCCG ATAGAAGAGA CATTGAGGCA
1711  ATTGTATGGG AAGATCATGA ACCAAAGACT TTTTGGGACA AATTTTGGAA TGTTGTAGCA
```

B.

1 ATGACAAATT CAAAAGAAGA CGCCGACATA GAGGAGAAGC ATATGTACAA TGAGCCGGTC ACAACCCTCT TTCACGACGT TGAAGCTTCA

91 CAAACACACC ACAGACGTGG GTCAATACCA TTAAAAGATG AGAAAAGTAA AGAATTGTAT CCATTGCGCT CTTTCCCGAC GAGAGTAAAT

181 GGCGAGGATA CGTTCTCTAT GGAGGATGGC ATAGGTGATG AAGATGAAGG AGAAGTACAG AACGCTGAAGT GAAGAGAGA GCTTA AGCAA

271 AGACATATTG GTATGATTGC CCTTGGTGGT ACTATTGGTA CAGGTCTTTT CATTGGTTTA TCCACACCTC TGACCAACGC CGGCCCAGTG

361 GGCGCTCTTA TATCATATTT ATTTATGGGT TCTTTGGCAT ATTCTGTCAC GCAGTCCTTG GGTGAAATGG CTACATTCAT CCCTGTTACA

451 TCCTCTTTCA CAGTGTTCTC ACAAAGATTC CTTTCTCCAG CATTTGGTGC GGCCAATGGT TACATGTATT GGTTTTCTTG GGCAATCACT

541 TTTGCCCTGG AACTTAGTGT GTTGGCCAA GTCATTCAAT TTTGGACGTA CAAAGTTCCA CTGGCGGCAT GGATTAGTAT TTTTTGGGTA

631 ATTATCACAA TAATGAACTT GTTCCCTGTC AAATATTACG GTGAATTCGA GTTCTGGGTC GCTTCCATCA AAGTTTTAGC CATTATCGGG

721 TTTCTAATAT ACTGTTTTTG TATGGTTTGT GGTGCTGGGG TTACCGGCCC AGTTGGATTC CGTTATTGGA GAAACCCAGG TGCCTGGGGT

811 CCAGGTATAA TATCTAAGGATAAAAACGAA GGGAGGTTCT TAGGTTGGGT TTCCTCTTTG ATTAACGCTG CCTTCACATT TCAAGGTACT

901 GAACTAGTTG GTATCACTGC TGGTGAAGCT GCAAACCCCA GAAAATCCGT TCCAAGAGCC ATCAAAAAAG TTGTTTTCCG TATCTTAACC

991 TTCTACATTG GCTCTCTATT ATTCATTGGA CTTTTAGTTC CATACAATGA CCCTAAACTA ACACAATCTA CTTCCTACGT TTCTACTTCT

1081 CCCTTTATTA TTGCTATTGA GAACTCTGGT ACAAAGGTTT TGCCACATAT CTTCAACGCT GTTATCTTAA CAACCATTAT TTCTGCCGCA

1171 AATTCAAATA TTTACGTTGG TTCCCGTATT TTATTTGGTC TATCAAAGAA CAAGTTGGCT CCTAAATTCC TGTCAAGGAC CACCAAAGGT

1261 GGTGTTCCAT ACATTGCAGT TTTCGTTACT GCTGCATTTG GCGCTTTGGC TTACATGGAG ACATCTACTG GTGGTGACAA AGTTTTCGAA

1351 TGGCTATTAA ATATCACTGG TGTTGCAGGC TTTTTTGCAT GGTTATTTAT CTCAATCTCG CACATCAGAT TTATGCAAGC TTTGAAATAC

1441 CGTGGCATCT CTCGTGACGA GTTACCATTT AAAGCTAAAT TAATGCCCGG CTTGGCTTAT TATGCGGCCA CATTTATGAC GATCATTATC

1531 ATTATTCAAG GTTTCACGGC TTTTGCACCA AAATTCAATG GTGTTAGCTT TGCTGCCGCC TATATCTCTG TTTTCCTGTT CTTAGCTGTT

1621 TGGATCTTAT TTCAATGCAT ATTCAGATGC AGATTTATTT GGAAGATTGG AGATGTCGAC ATCGATTCCG ATAGAAGAGA CATTGAGGCA

1711 ATTGTATGGG AAGATCATGA ACCAAAGACT TTTTGGGACA AATTTTGGAA TGTTGTAGCA

C.

```
1     ATGACAAATT CAAAAGAAGA CGCCGACATA GAGGAGAAGC ATATGTACAA TGAGCCGGTC ACAACCCTCT TTCACGACGT TGAAGCTTCA
91    CAAACACACC ACAGACGTGG GTCAATACCA TTAAAAGATG AGAAAAGTAA AGAATTGTAT CCATTGCGCT CTTTCCCGAC GAGAGTAAAT
181   GGCGAGGATA CGTTCTCTAT GGAGGATGGC ATAGGTGATG AAGATGAAGG AGAAGTACAG AACGCTGAAG TGAAGAGAGA GCTTAAGCAA
271   AGACATATTG GTATGATTGC CCTTGGTGGT ACTATTGGTA CAGGTCTTTT CATTGGTTTA TCCACACCTC TGACCAACGC CGGCCCAGTG
361   GGCGCTCTTA TATCATATTT ATTTATGGGT TCTTTGGCAT ATTCTGTCAC GCAGTCCTTG GGTGAAATGG CTACATTCAT CCCTGTTACA
451   TCCTCTTTCA CAGTGTTCTC ACAAAGATTC CTTTCTCCAG CATTTGGTGC GGCCAATGGT TACATGTATT GGTTTTCTTG GGCAATCACT
541   TTTGCCCTGG AACTTAGTGT GTTGGCCAA GTCATTCAAT TTTGGACGTA CAAAGTTCCA CTGGCGGCAT GGATTAGTAT TTTTTGGGTA
631   ATTATCACAA TAATGAACTT GTTCCCTGTC AAATATTACG GTGAATTCGA GTTCTGGGTC GCTTCCATCA AAGTTTTAGC CATTATCGGG
721   TTTCTAATAT ACTGTTTTTG TATGGTTTGT GGTGCTGGGG TTACCGGCCC AGTTGGATTC CGTTATTGGA GAAACCCAGG TGCCTGGGGT
811   CCAGGTATAA TATCTAAGGA TAAAAACGAA GGGAGGTTCT TAGGTTGGGT TTCCTCTTTG ATTAACGCTG CCTTCACATT TCAAGGTACT
901   GAACTAGTTG GTATCACTGC TGGTGAAGCT GCAAACCCCA GAAAATCCGT TCCAAGAGCC ATCAAAAAAG TTGTTTTCCG TATCTTAACC
991   TTCTACATTG GCTCTCTATT ATTCATTGGA CTTTTAGTTC CATACAATGA CCCTAAACTA ACACAATCTA CTTCCTACGT TTCTACTTCT
1081  CCCTTTATTA TTGCTATTGA GAACTCTGGT ACAAAGGTTT TGCCACATAT CTTCAACGCT GTTATCTTAA CAACCATTAT TTCTGCCGCA
1171  AATTCAAATA TTTACGTTGG TTCCCGTATT TTATTTGGTC TATCAAAGAA CAAGTTGGCT CCTAAATTCC TGTCAAGGAC CACCAAAGGT
1261  GGTGTTCCAT ACATTGCAGT TTTCGTTACT GCTGCATTTG GCGCTTTGGC TTACATGGAG ACATCTACTG GTGGTGACAA AGTTTTCGAA
1351  TGGCTATTAA ATATCACTGG TGTTGCAGGC TTTTTTGCAT GGTTATTTAT CTCAATCTCG CACATCAGAT TTATGCAAGC TTTGAAATAC
1441  CGTGGCATCT CTCGTGACGA GTTACCATTT AAAGCTAAAT TAATGCCCGG CTTGGCTTAT TATGCGGCCA CATTTATGAC GATCATTATC
1531  ATTATTCAAG GTTTCACGGC TTTTGCACCA AAATTCAATG GTGTTAGCTT TGCTGCCGCC TATATCTCTG TTTTCCTGTT CTTAGCTGTT
1621  TGGATCTTAT TTCAATGCAT ATTCAGATGC AGATTTATTT GGAAGATTGG AGATGTCGAC ATCGATTCCG ATAGAAGAGA CATTGAGGCA
1711  ATTGTATGGG AAGATCATGA ACCAAAGACT TTTTGGGACA AATTTTGGAA TGTTGTAGCA
```

Supplemental Figure S1: Mutational Spectra of wild-type and *rev3*-Δ*C* mapped to *CAN1* gene

Black continuous text represents the sequence of the *CAN1* gene in *Saccharomyces cerevisiae*. Black text/bases above *CAN1* represent mutations found in wild-type clones and blue text/bases below *CAN1* indicate mutations found in *rev3*-Δ*C* clones. Red boxes encase wild-type complex mutations and green boxes encase *rev3*-Δ*C* complex mutations. Triangles represent deletions, if a number is present it indicates how many bases were deleted otherwise it was one base. Red bases in the *CAN1* sequence are polymorphisms found in the 8C strain that differ from the reference strain deposited in the *Saccharomyces* Genome Database. **A.** 20 J/m^2 **B.** 40 J/m^2 **C.** 60 J/m^2
