# Supplementary material for: DNA Polymerase ζ without the C-Terminus of Catalytic Subunit Rev3 Retains Characteristic Activity, but Alters Mutation Specificity of Ultraviolet Radiation in Yeast
Source: Genes (Basel). 2022 Sep 2;13(9):1576. doi: 10.3390/genes13091576 (PMC9498848; doi:10.3390/genes13091576)
Supplement: Supplementary file 1 [file genes-13-01576-s001.zip › Suppl Figure S2.pdf]

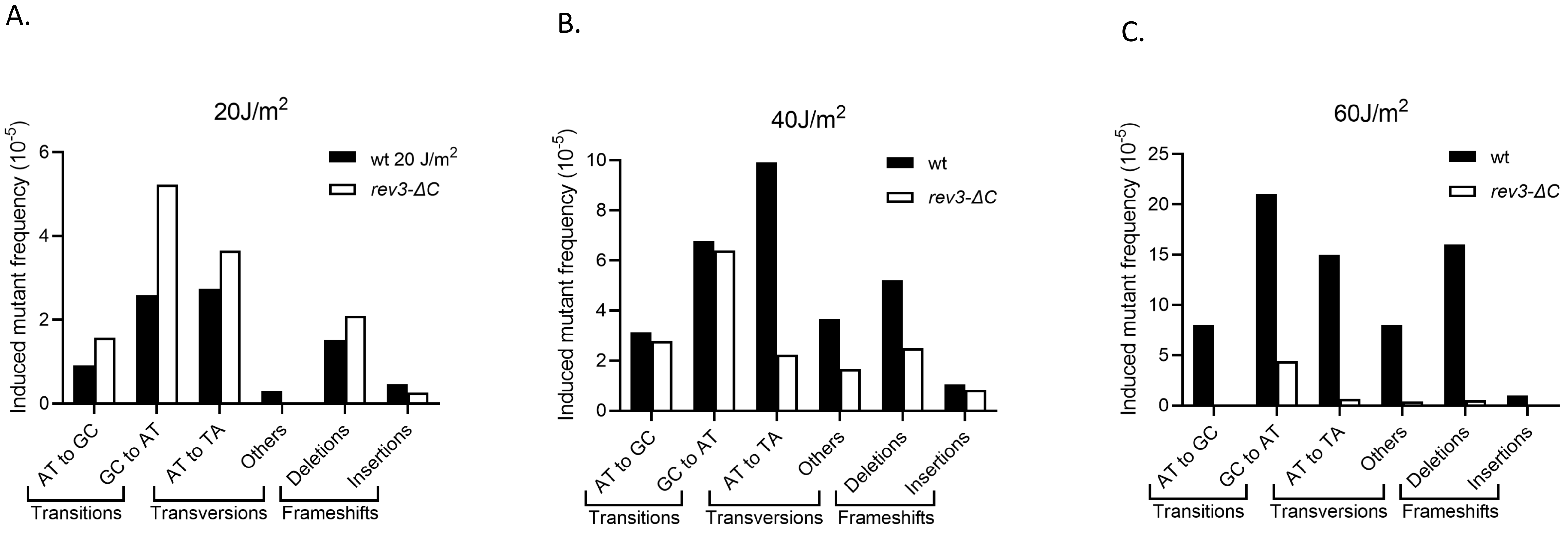

Supplemental Figure S2. UV dose-dependent changes in frequencies of transition, transversion and indel mutations in the wild-type and *rev3-ΔC* strains.
