# Supplementary material for: DNA Polymerase ζ without the C-Terminus of Catalytic Subunit Rev3 Retains Characteristic Activity, but Alters Mutation Specificity of Ultraviolet Radiation in Yeast
Source: Genes (Basel). 2022 Sep 2;13(9):1576. doi: 10.3390/genes13091576 (PMC9498848; doi:10.3390/genes13091576)
Supplement: Supplementary file 1 [file genes-13-01576-s001.zip › Suppl Table S1.pdf]

Supplemental Table S1. Differences in classes of mutations in wild-type and *rev3-ΔC* strains

Mutation type	UV dose (J/m^2)								
	20			40			60		
	Wild-type	*rev3-ΔC*	*P* value#	Wild-type	*rev3-ΔC*	*P* value#	Wild-type	*rev3-ΔC*	*P* value#
**Single base change**	37	43	0.0700	42	45	0.7845	41	45	0.0858
Rate*	6.7	12.0		25.0	14.2		51.5	5.5	
Fold decrease##			0.6			1.8			9.3
**Insertions/deletions >1nt**	2	0	0.4946	1	1	1.0000	2	0	0.4960
Rate*	0.4	0		0.6	0.3		2.5	0	
Fold decrease			-			2.0			-
**Tandem double**	1	2	0.6168	1	2	1.0000	4	5	0.7353
Rate*	0.2	0.6		0.6	0.6		5.0	0.6	
Fold decrease			0.3			1.0			8.3
**Complex###**	7	1	0.0588	6	4	0.5214	8	1	0.0324
Rate*	1.3	0.3		3.6	1.3		10.0	0.1	
Fold decrease			4.3			2.8			100

Total changes	47	46		50	52		55	51	
Total rate*	8.5	12.8		29.7	16.4		69.0	6.25	
Fold decrease			0.7			1.8			11.1

*-Induced mutant frequency x 10^{-5}.

#-Fisher's exact test.

##-Fold decrease of induced mutant frequency in *rev3-ΔC* comparing to wild-type.

###-Tandem double excluded.
