# Supplementary material for: DNA Polymerase ζ without the C-Terminus of Catalytic Subunit Rev3 Retains Characteristic Activity, but Alters Mutation Specificity of Ultraviolet Radiation in Yeast
Source: Genes (Basel). 2022 Sep 2;13(9):1576. doi: 10.3390/genes13091576 (PMC9498848; doi:10.3390/genes13091576)
Supplement: Supplementary file 1 [file genes-13-01576-s001.zip › Suppl Table S2.pdf]

Supplemental Table S2. Differences in mutation spectra in wild-type and *rev3-ΔC* strains

Mutation type*	UV dose (J/m^2)								
	20			40			60		
	Wild-type	*rev3-ΔC*	*P* value#	Wild-type	*rev3-ΔC*	*P* value#	Wild-type	*rev3-ΔC*	*P* value#
**Transitions**									
AT to GC	6	6	0.0638	6	10		8	**1#**	0.0386
GC to AT	17	20	0.3087	13	**23#**	0.0052	21	**41#**	<0.0001
Total	23	26	0.2442	19	**33#**	0.0054	29	**42#**	0.0007
Rate**	3.5	6.8		9.9	9.1		29.0	4.5	
Fold decrease##			0.5			1.1			6.4
**Transversions**									
AT to TA	18	14	0.8321	19	**8#**	0.0206	15	6	0.0981
Other	2	0	0.4974	7	6		8	4	0.5444
Total	20	14	0.5317	26	**14#**	0.0132	23	**10#**	0.0445
Rate**	3.0	3.7		13.6	3.9		24.0	1.1	
Fold decrease##			0.8			3.5			22.3
**Frameshifts**									
Deletions	10	8	1.0000	10	9		16	**5#**	0.0322
Insertions	3	1	0.6211	2	3		1	1	1.0000

Total	13	9	0.6339	12	12		17	**6**[#]	0.0407
Rate**	2.0	2.4		6.2	3.3		16.0	0.6	
Fold decrease[##]			0.8			1.9			24.8
Total changes	56	49		57	59		69	58	
Total rate**	8.5	12.8		29.7	16.4		69.0	6.25	
Fold decrease[##]			0.7			1.8			11.1

*-Substitutions found in mutants double tandem mutants are counted as individual single base substitution.

**-Induced mutant frequency x 10^{-5}.

[#]-Exact values are given; statistically significant difference in proportion of mutations are in **BOLD**.

[##]-Fold decrease of induced mutant frequency in *rev3-ΔC* comparing to wild-type.
